# Opposite trends of glycosides and alkaloids in *Dendrobium nobile* of different age based on UPLC‐Q/TOF‐MS combined with multivariate statistical analyses

**DOI:** 10.1002/pca.3115

**Published:** 2022-03-02

**Authors:** An‐jing Lu, Yuan Jiang, Jia Wu, Dao‐peng Tan, Lin Qin, Yan‐liu Lu, Yong Qian, Chao‐jun Bai, Ji‐yong Yang, Hua Ling, Jing‐shan Shi, Zhou Yang, Yu‐qi He

**Affiliations:** ^1^ Key Laboratory of Basic Pharmacology of Ministry of Education and Joint International Research Laboratory of Ethnomedicine of Ministry of Education, School of Pharmacy Zunyi Medical University Zunyi Guizhou China; ^2^ Shanghai Standard Technology Co., Ltd Shanghai China; ^3^ Guangxi Shenli Pharmaceutical Co., Ltd. Yulin Guangxi China; ^4^ Chishui Xintian Chinese Medicine Industry Development Co., Ltd Zunyi Guizhou China; ^5^ School of Pharmacy Georgia Campus ‐ Philadelphia College of Osteopathic Medicine Suwanee GA USA

**Keywords:** alkaloids, *Dendrobium nobile* Lindl., glycosides, PLS‐DA, UPLC‐Q/TOF‐MS

## Abstract

**Introduction:**

Alkaloids and glycosides are the active ingredients of the herb *Dendrobium nobile*, which is used in traditional Chinese medicine. The pharmacological effects of alkaloids include neuroprotective effects and regulatory effects on glucose and lipid metabolism, while glycosides improve the immune system. The pharmacological activities of the above chemical components are significantly different. In practice, the stems of 3‐year‐old *D. nobile* are usually used as the main source of Dendrobii Caulis. However, it has not been reported whether this harvesting time is appropriate.

**Objective:**

The aim of this study was to compare the chemical characteristics of *D. nobile* in different growth years (1–3 years).

**Methods:**

In this study, ultra‐high‐performance liquid chromatography coupled with quadrupole time‐of‐flight tandem mass spectrometry (UPLC‐Q/TOF‐MS) was employed to analyze the constituents of *D. nobile*. The relative abundance of each constituent was analyzed with multivariate statistical analyses to screen the characteristic constituents that contributed to the characterization and classification of *D. nobile*. Dendrobine, a component of *D. nobile* that is used for quality control according to the Chinese Pharmacopoeia, was assayed by gas chromatography.

**Results:**

As a result, 34 characteristic constituents (VIP > 2) were identified or tentatively identified as alkaloids and glycosides based on MS/MS data. Moreover, the content of alkaloids decreased over time, whereas the content of glycosides showed the opposite trend. The absolute quantification of dendrobine was consistent with the metabolomics results.

**Conclusion:**

Our findings provide valuable information to optimize the harvest period and a reference for the clinical application of *D. nobile*.

## Abstract 2

In this work, the chemical characteristics of *D. nobile* with different growth years were determined using UPLC‐Q/TOF‐MS coupled with multivariate statistical analyses. The results showed the opposite accumulation trends of glycosides and alkaloids in *D. nobile* with different growth years. The above results may have been caused by an underlying mechanism. In summary, the new findings could provide valuable information for the optimum harvest period and provide reference for clinical drug therapeutics.

## 1 INTRODUCTION

*Dendrobium* is the second largest genus of the plant family of Orchidaceae. Approximately 1,500 *Dendrobium* species are currently known.^1^ Dendrobii Caulis, Ganoderma, Cordyceps, and Ginseng Radix et Rhizoma are honored as “superior‐grade” herbs in an ancient book named “*Shen nong's Classic of Materia Medica*,” which is one of the Four Classics of traditional Chinese medicine. Moreover, Dendrobii Caulis has the effects of nourishing Yin and clearing heat, and it is applicable to situations such as yin injury and depletion of body fluid, dry mouth and polydipsia, less food and retching, deficiency and heat after illness, and dark eye.^2^

In the Chinese Pharmacopeia, *D. nobile* is listed as the primary medicinal source of Dendrobii Caulis.^3^ Pharmacological research confirmed that *D. nobile* improves cognitive dysfunction, reduces gastric damage, has anti‐tumor effects, and can be used to treat diabetes.^4^ Due to its beneficial effects, *D. nobile* is also used as a tonic and functional food, such as Dendrobii Liquor.^5^ Broad applications increased the demand for *D. nobile* and hence resulted in a sharp decline of wild *D. nobile* populations. In the Convention on International Trade in Endangered Species of Wild Fauna and Flora (CITES) Appendix II, wild *D. nobile* is included.^6^

To meet the demands, *D. nobile* was developed. The alkaloids and glycosides of *D. nobile* are considered as the principal biologically active components with extensive pharmacological effects. The alkaloids are used to protect neurons and regulate glucose and lipid metabolism, among others.^7^ Dendromoniliside A, isolated from the stem of *Dendrobium moniliforme*, improves immune activity by stimulating the proliferation of B cells and inhibiting the proliferation of T cells in vitro.^8^ Dendronobiloside A, isolated from the stem of *D. nobile*, was found to stimulate the proliferation of murine T and B lymphocytes in vitro.^9^, ^10^ The above compounds have different pharmacological effects. In the main producing region, the stems of *D. nobile* are usually harvested in the third year. However, little evidence is available to support this practice. To find out the best time to harvest, understanding the differences in metabolite profile between *D. nobile* grown for various years is necessary.

Fourteen alkaloids, including dendrobine, dendrobine‐*N*‐oxide, dobilonine, and dendroxine, were isolated from *D. nobile* in previous studies.^11^, ^12^ Among them, dendrobine is used as a quality marker for the quality control (QC) of *D. nobile* according to the Chinese Pharmacopeia. However, our previous study indicated that the content of dendrobine in *D. nobile* decreases over time. Moreover, it is unknown whether other alkaloids in *D. nobile* follow the same trend as dendrobine. In addition to alkaloids, some non‐alkaloid chemical constituents, such as polysaccharides, sesquiterpenes, phenanthrene, bibenzyl, and fluorenones, also exist in *D. nobile*.^13^ How the levels of these non‐alkaloid chemical constituents in *D. nobile* change with age is also unclear. Therefore, it is unclear whether dendrobine should be used as a quality marker for the QC of *D. nobile*.

In the present study, ultra‐high‐performance liquid chromatography coupled with quadrupole time‐of‐flight tandem mass spectrometry (UPLC‐Q/TOF‐MS) coupled with multivariate statistical analysis was employed to probe the characteristic components of *D. nobile* in different growth years to find accurate quality markers in *D. nobile*.

## 2 EXPERIMENTAL PROCEDURES

### 2.1 Chemicals and reagents

Dendrobine (purity > 99%) was purchased from the National Institutes for Food and Drug Control (Beijing, China). Naphthalene (purity > 99%, internal standard [IS]) was purchased from Sigma‐Aldrich (St. Louis, MO, USA). LC‐MS‐grade acetonitrile and methanol were supplied by Merck (Darmstadt, Germany). LC‐MS‐grade formic acid was purchased from CNW Technologies GmbH (Dusseldorf, Germany). Formic acid and methanol (AR) were obtained from Chengdu Kelong Chemical Co., Ltd. (Chengdu, China).

### 2.2 Sample collection

The fresh stems of 1‐ to 3‐year‐old *D. nobile* were harvested from Chishui city of Guizhou Province in China in October 2019. Samples were collected in triplicate for each feature. Details are listed in Table 1.

**TABLE 1:** The information of *D. nobile* samples

Sample number	Cluster number	Stem number	Growth year	Collection time	Origin	Species of epiphytic stone
2019‐10‐10–1‐1	2019‐10‐10–1	1	1 year	2019‐10‐10	Chishui city, Guizhou Province	Danxia Stone
2019‐10‐10–1‐2	2019‐10‐10–1	2	1 year	2019‐10‐10	Chishui city, Guizhou Province	Danxia Stone
2019‐10‐10–1‐3	2019‐10‐10–1	3	1 year	2019‐10‐10	Chishui city, Guizhou Province	Danxia Stone
2019‐10‐10–1‐4	2019‐10‐10–1	4	2 years	2019‐10‐10	Chishui city, Guizhou Province	Danxia Stone
2019‐10‐10–1‐5	2019‐10‐10–1	5	2 years	2019‐10‐10	Chishui city, Guizhou Province	Danxia Stone
2019‐10‐10–1‐6	2019‐10‐10–1	6	2 years	2019‐10‐10	Chishui city, Guizhou Province	Danxia Stone
2019‐10‐10–1‐7	2019‐10‐10–1	7	3 years	2019‐10‐10	Chishui city, Guizhou Province	Danxia Stone
2019‐10‐10–1‐8	2019‐10‐10–1	8	3 years	2019‐10‐10	Chishui city, Guizhou Province	Danxia Stone
2019‐10‐10–1‐9	2019‐10‐10–1	9	3 years	2019‐10‐10	Chishui city, Guizhou Province	Danxia Stone
2019‐10‐10–2‐1	2019‐10‐10–2	1	1 year	2019‐10‐10	Chishui city, Guizhou Province	Danxia Stone
2019‐10‐10–2‐2	2019‐10‐10–2	2	1 year	2019‐10‐10	Chishui city, Guizhou Province	Danxia Stone
2019‐10‐10–2‐3	2019‐10‐10–2	3	1 year	2019‐10‐10	Chishui city, Guizhou Province	Danxia Stone
2019‐10‐10–2‐4	2019‐10‐10–2	4	2 years	2019‐10‐10	Chishui city, Guizhou Province	Danxia Stone
2019‐10‐10–2‐5	2019‐10‐10–2	5	2 years	2019‐10‐10	Chishui city, Guizhou Province	Danxia Stone
2019‐10‐10–2‐6	2019‐10‐10–2	6	2 years	2019‐10‐10	Chishui city, Guizhou Province	Danxia Stone
2019‐10‐10–2‐7	2019‐10‐10–2	7	3 years	2019‐10‐10	Chishui city, Guizhou Province	Danxia Stone
2019‐10‐10–2‐8	2019‐10‐10–2	8	3 years	2019‐10‐10	Chishui city, Guizhou Province	Danxia Stone
2019‐10‐10–2‐9	2019‐10‐10–2	9	3 years	2019‐10‐10	Chishui city, Guizhou Province	Danxia Stone
2019‐10‐10–3‐1	2019‐10‐10–3	1	1 year	2019‐10‐10	Chishui city, Guizhou Province	Danxia Stone
2019‐10‐10–3‐2	2019‐10‐10–3	2	1 year	2019‐10‐10	Chishui city, Guizhou Province	Danxia Stone
2019‐10‐10–3‐3	2019‐10‐10–3	3	1 year	2019‐10‐10	Chishui city, Guizhou Province	Danxia Stone
2019‐10‐10–3‐4	2019‐10‐10–3	4	2 years	2019‐10‐10	Chishui city, Guizhou Province	Danxia Stone
2019‐10‐10–3‐5	2019‐10‐10–3	5	2 years	2019‐10‐10	Chishui city, Guizhou Province	Danxia Stone
2019‐10‐10–3‐6	2019‐10‐10–3	6	2 years	2019‐10‐10	Chishui city, Guizhou Province	Danxia Stone
2019‐10‐10–3‐7	2019‐10‐10–3	7	3 years	2019‐10‐10	Chishui city, Guizhou Province	Danxia Stone
2019‐10‐10–3‐8	2019‐10‐10–3	8	3 years	2019‐10‐10	Chishui city, Guizhou Province	Danxia Stone
2019‐10‐10–4‐1	2019‐10‐10–4	1	1 year	2019‐10‐10	Chishui city, Guizhou Province	Danxia Stone
2019‐10‐10–4‐2	2019‐10‐10–4	2	1 year	2019‐10‐10	Chishui city, Guizhou Province	Danxia Stone
2019‐10‐10–4‐3	2019‐10‐10–4	3	1 year	2019‐10‐10	Chishui city, Guizhou Province	Danxia Stone
2019‐10‐10–4‐4	2019‐10‐10–4	4	2 years	2019‐10‐10	Chishui city, Guizhou Province	Danxia Stone
2019‐10‐10–4‐5	2019‐10‐10–4	5	2 years	2019‐10‐10	Chishui city, Guizhou Province	Danxia Stone
2019‐10‐10–4‐6	2019‐10‐10–4	6	2 years	2019‐10‐10	Chishui city, Guizhou Province	Danxia Stone
2019‐10‐10–4‐7	2019‐10‐10–4	7	3 years	2019‐10‐10	Chishui city, Guizhou Province	Danxia Stone
2019‐10‐10–4‐8	2019‐10‐10–4	8	3 years	2019‐10‐10	Chishui city, Guizhou Province	Danxia Stone
2019‐10‐10–4‐9	2019‐10‐10–4	9	3 years	2019‐10‐10	Chishui city, Guizhou Province	Danxia Stone
2019‐10‐10–5‐1	2019‐10‐10–5	1	1 year	2019‐10‐10	Chishui city, Guizhou Province	Danxia Stone
2019‐10‐10–5‐2	2019‐10‐10–5	2	1 year	2019‐10‐10	Chishui city, Guizhou Province	Danxia Stone
2019‐10‐10–5‐3	2019‐10‐10–5	3	1 year	2019‐10‐10	Chishui city, Guizhou Province	Danxia Stone
2019‐10‐10–5‐4	2019‐10‐10–5	4	2 years	2019‐10‐10	Chishui city, Guizhou Province	Danxia Stone
2019‐10‐10–5‐5	2019‐10‐10–5	5	2 years	2019‐10‐10	Chishui city, Guizhou Province	Danxia Stone
2019‐10‐10–5‐6	2019‐10‐10–5	6	2 years	2019‐10‐10	Chishui city, Guizhou Province	Danxia Stone
2019‐10‐10–5‐7	2019‐10‐10–5	7	3 years	2019‐10‐10	Chishui city, Guizhou Province	Danxia Stone
2019‐10‐10–5‐8	2019‐10‐10–5	8	3 years	22019‐10‐10	Chishui city, Guizhou Province	Danxia Stone
2019‐10‐10–5‐9	2019‐10‐10–5	9	3 years	2019‐10‐10	Chishui city, Guizhou Province	Danxia Stone
2019‐10‐10–6‐1	2019‐10‐10–6	1	1 year	2019‐10‐10	Chishui city, Guizhou Province	Danxia Stone
2019‐10‐10–6‐2	2019‐10‐10–6	2	1 year	2019‐10‐10	Chishui city, Guizhou Province	Danxia Stone
2019‐10‐10–6‐3	2019‐10‐10–6	3	1 year	2019‐10‐10	Chishui city, Guizhou Province	Danxia Stone
2019‐10‐10–6‐4	2019‐10‐10–6	4	2 years	2019‐10‐10	Chishui city, Guizhou Province	Danxia Stone
2019‐10‐10–6‐5	2019‐10‐10–6	5	2 years	2019‐10‐10	Chishui city, Guizhou Province	Danxia Stone
2019‐10‐10–6‐6	2019‐10‐10–6	6	2 years	2019‐10‐10	Chishui city, Guizhou Province	Danxia Stone
2019‐10‐10–6‐7	2019‐10‐10–6	7	3 years	2019‐10‐10	Chishui city, Guizhou Province	Danxia Stone
2019‐10‐10–6‐8	2019‐10‐10–6	8	3 years	2019‐10‐10	Chishui city, Guizhou Province	Danxia Stone
2019‐10‐10–6‐9	2019‐10‐10–6	9	3 years	2019‐10‐10	Chishui city, Guizhou Province	Danxia Stone
2019‐10‐10–7‐1	2019‐10‐10–7	1	1 year	2019‐10‐10	Chishui city, Guizhou Province	Danxia Stone
2019‐10‐10–7‐2	2019‐10‐10–7	2	1 year	2019‐10‐10	Chishui city, Guizhou Province	Danxia Stone
2019‐10‐10–7‐3	2019‐10‐10–7	3	1 year	2019‐10‐10	Chishui city, Guizhou Province	Danxia Stone
2019‐10‐10–7‐4	2019‐10‐10–7	4	2 years	2019‐10‐10	Chishui city, Guizhou Province	Danxia Stone
2019‐10‐10–7‐5	2019‐10‐10–7	5	2 years	2019‐10‐10	Chishui city, Guizhou Province	Danxia Stone
2019‐10‐10–7‐6	2019‐10‐10–7	6	2 years	2019‐10‐10	Chishui city, Guizhou Province	Danxia Stone
2019‐10‐10–7‐7	2019‐10‐10–7	7	3 years	2019‐10‐10	Chishui city, Guizhou Province	Danxia Stone
2019‐10‐10–7‐8	2019‐10‐10–7	8	3 years	2019‐10‐10	Chishui city, Guizhou Province	Danxia Stone
2019‐10‐10–7‐9	2019‐10‐10–7	9	3 years	2019‐10‐10	Chishui city, Guizhou Province	Danxia Stone
2019‐10‐10–7‐10	2019‐10‐10–7	10	3 years	2019‐10‐10	Chishui city, Guizhou Province	Danxia Stone
2019‐10‐10–8‐1	2019‐10‐10–8	1	1 year	2019‐10‐10	Chishui city, Guizhou Province	Danxia Stone
2019‐10‐10–8‐2	2019‐10‐10–8	2	1 year	2019‐10‐10	Chishui city, Guizhou Province	Danxia Stone
2019‐10‐10–8‐3	2019‐10‐10–8	3	1 year	2019‐10‐10	Chishui city, Guizhou Province	Danxia Stone
2019‐10‐10–8‐4	2019‐10‐10–8	4	2 years	2019‐10‐10	Chishui city, Guizhou Province	Danxia Stone
2019‐10‐10–8‐5	2019‐10‐10–8	5	2 years	2019‐10‐10	Chishui city, Guizhou Province	Danxia Stone
2019‐10‐10–8‐6	2019‐10‐10–8	6	2 years	2019‐10‐10	Chishui city, Guizhou Province	Danxia Stone
2019‐10‐10–8‐7	2019‐10‐10–8	7	3 years	2019‐10‐10	Chishui city, Guizhou Province	Danxia Stone
2019‐10‐10–8‐8	2019‐10‐10–8	8	3 years	2019‐10‐10	Chishui city, Guizhou Province	Danxia Stone
2019‐10‐10–8‐9	2019‐10‐10–8	9	3 years	2019‐10‐10	Chishui city, Guizhou Province	Danxia Stone
2019‐10‐10–9‐1	2019‐10‐10–9	1	1 year	2019‐10‐10	Chishui city, Guizhou Province	Danxia Stone
2019‐10‐10–9‐2	2019‐10‐10–9	2	1 year	2019‐10‐10	Chishui city, Guizhou Province	Danxia Stone
2019‐10‐10–9‐3	2019‐10‐10–9	3	1 year	2019‐10‐10	Chishui city, Guizhou Province	Danxia Stone
2019‐10‐10–9‐4	2019‐10‐10–9	4	2 years	2019‐10‐10	Chishui city, Guizhou Province	Danxia Stone
2019‐10‐10–9‐5	2019‐10‐10–9	5	2 years	2019‐10‐10	Chishui city, Guizhou Province	Danxia Stone
2019‐10‐10–9‐6	2019‐10‐10–9	6	2 years	2019‐10‐10	Chishui city, Guizhou Province	Danxia Stone
2019‐10‐10–9‐7	2019‐10‐10–9	7	3 years	2019‐10‐10	Chishui city, Guizhou Province	Danxia Stone
2019‐10‐10–9‐8	2019‐10‐10–9	8	3 years	2019‐10‐10	Chishui city, Guizhou Province	Danxia Stone
2019‐10‐10–9‐9	2019‐10‐10–9	9	3 years	2019‐10‐10	Chishui city, Guizhou Province	Danxia Stone
2019‐10‐10–10‐1	2019‐10‐10–10	1	1 year	2019‐10‐10	Chishui city, Guizhou Province	Danxia Stone
2019‐10‐10–10‐2	2019‐10‐10–10	2	1 year	2019‐10‐10	Chishui city, Guizhou Province	Danxia Stone
2019‐10‐10–10‐3	2019‐10‐10–10	3	1 year	2019‐10‐10	Chishui city, Guizhou Province	Danxia Stone
2019‐10‐10–10‐4	2019‐10‐10–10	4	2 years	2019‐10‐10	Chishui city, Guizhou Province	Danxia Stone
2019‐10‐10–10‐5	2019‐10‐10–10	5	2 years	2019‐10‐10	Chishui city, Guizhou Province	Danxia Stone
2019‐10‐10–10‐6	2019‐10‐10–10	6	2 years	2019‐10‐10	Chishui city, Guizhou Province	Danxia Stone
2019‐10‐10–10‐7	2019‐10‐10–10	7	3 years	2019‐10‐10	Chishui city, Guizhou Province	Danxia Stone
2019‐10‐10–10‐8	2019‐10‐10–10	8	3 years	2019‐10‐10	Chishui city, Guizhou Province	Danxia Stone
2019‐10‐10–10‐9	2019‐10‐10–10	9	3 years	2019‐10‐10	Chishui city, Guizhou Province	Danxia Stone

### 2.3 Sample preparation to profile the metabolome of *D. nobile* by UPLC‐Q/TOF‐MS

Clean fresh *D. nobile* stems were dried at 60°C in a drying oven. The dried stems were ground into fine powder (300 mesh), and then 75 mg was soaked in 1 mL of 70% (v/v) methanol, followed by ultrasonication (50 kHz, 400 W) for 30 min. Next, the liquid was centrifuged at 9705 g  for 5 min, and the supernatant was used for UPLC‐Q/TOF‐MS analysis.

### 2.4 UPLC/Q‐TOF/MS parameters

A 1290 Infinity II UPLC system (Agilent, MA, USA) coupled with a Synapt Q/TOF‐MS System (Agilent, MA, USA) was used to profile the metabolome of *D. nobile*. Sample solution aliquots of 0.1 μL were separated on a Waters CORTECS UPLC C18 column (100 mm × 2.1 mm, 1.6 μm) at 40°C with a flow rate of 0.4 mL/min. The mobile phases consisted of solvent A (0.1% formic acid in water, v/v) and solvent B (0.1% formic acid in acetonitrile, v/v). The elution gradients were as follows: 0–0.5 min, 5% B; 0.5–4 min, 5%–40% B; 4–5 min, 40%–75% B; 5–5.1 min, 75%–95% B; 5.1–6.5 min, 95% B; 6.5–6.6 min, 95%–5% B; 6.6–10 min, 5% B. Electronic spray ionization (ESI) was used in the TOF‐MS system. The ion source temperature was 350°C, the nebulizer pressure was 44 psi, the drying gas flow was 10 L/min, the sheath gas flow temperature was 350°C, the sheath gas flow was 11 L/min, the Vcap voltage was 4,000 V, and the nozzle voltage was 1,000 V. In the mass analyzer, the scan mode was auto MS/MS, and the ions were scanned from *m*/*z* 50 to 1,200. [Correction added on 20 April 2022, after first online publication: In the first sentence under ‘2.4 UPLC/Q‐TOF/MS parameters’, the text “Waters Corp., Milford, MA, USA” has been changed to “Agilent, MA, USA”; and the elution gradients have been changed to "0–0.5 min, 5% B; 0.5–4 min, 5%–40% B; 4–5 min, 40%–75% B; 5–5.1 min, 75%–95% B; 5.1–6.5 min, 95% B; 6.5–6.6 min, 95%–5% B; 6.6–10 min, 5% B.”.]

### 2.5 Dendrobine quantification in *D. nobile* by GC

Dendrobine in *D. nobile* was quantified following Part I of the Chinese Pharmacopeia, edition 2015. Chromatography was performed on an Agilent gas chromatograph (Agilent Technologies 7820, USA) equipped with a DB‐1 column (30 m × 0.25 mm, 0.25 μm, Agilent Technologies). The linearity, precision, reproducibility, and stability were validated for dendrobine quantification.

### 2.6 Establishment of the in‐house library for *D. nobile*

To ensure the efficiency and accuracy of UPLC‐HRMS chemical profiling, an in‐house chemical library was constructed by retrieving several databases, including Scifinder (https://scifinder.cas.org/), Web of Science (http://www.webofscience.com), PubMed (https://pubmed.ncbi.nlm.nih.gov/), ChemSpider (http://www.chemspider.com/), and PubChem (https://pubchem.ncbi.nlm.nih.gov/). More than 700 references about *Dendrobium* Sw. were utilized to build the library including 430 compounds, which involved as much prior knowledge and available chemical information as possible, such as Chinese and English names, detailed molecular formulae, molecular weight, and CAS numbers in a Microsoft Office Excel document, as well as chemical structures in MOL files. Through use of the “Find” function of Excel, those compounds which matched the determined molecular formula were found as potential candidates. Only those metabolite data which further matched the experimental UPLC‐Q/TOF‐MS/MS data were finally selected for establishing the identity of an unknown constituent.

### 2.7 Data processing

The raw data were processed by peak matching, peak alignment, ion fusion, and deconvolution using Agilent Mass Hunter Profinder software (version 10.0). Principle component analysis (PCA) and partial least squares discriminant analysis (PLS‐DA) were performed in SIMCA‐P software (version 14.0). Other visualizations were performed in R (version 4.0.2). The statistical analysis of multiple comparisons was performed using one‐way ANOVA in SPSS 18.0 (IBM, Chicago, USA). Data in histograms and box plots are expressed as mean ± SD. *P*‐values of less than 0.05 were considered statistically significant.

## 3 RESULTS AND DISCUSSION

### 3.1 Profiling of the metabolome in *D. nobile* stems with different ages

**FIGURE 1:**
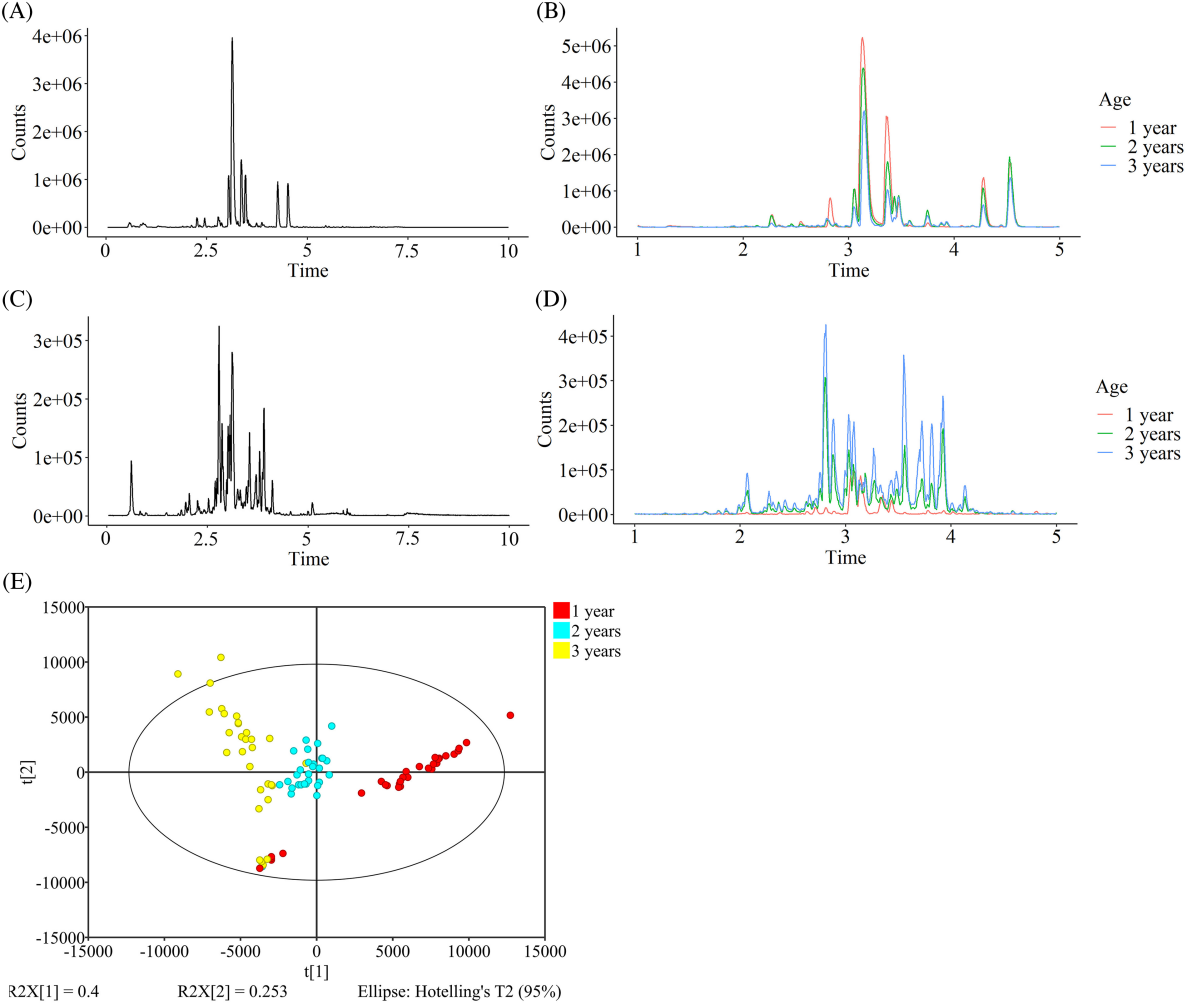
Effect of growth age on the metabolome of *D. nobile* stems. (A) The BPC of the positive mode of the QC sample. (B) The BPC of the positive mode of the stem samples of typical 1‐ to 3‐year‐old *D. nobile*. (C) The BPC of the negative mode of the QC sample. (D) The BPC of the negative mode of the stem samples of typical 1‐ to 3‐year‐old *D. nobile*. (E) Score plots of PCA of metabolome profiles (R2X (cum) = 0.721, Q2 (cum) = 0.650). Each spot represents a *D. nobile* sample

In the present study, the metabolites in *D. nobile* stems were separated by the UPLC system and detected in both positive and negative modes of Q/TOF‐MS. The total ion chromatograms are shown as the style of the base peak chromatograms (BPCs) for typical *D. nobile* samples cultivated for 1, 2, and 3 years. In the BPCs of both positive and negative modes, chromatographic peaks mainly appeared at the retention time range from 1 to 5 min (Figure 1A, C). In the BPC of the positive mode, a couple of peaks showed decreasing trends along with the increase of growth years (Figure 1B). Oppositely, in the BPC of the negative mode, another set of peak responses increased along with the growth years of the plants (Figure 1D).

Moreover, in total 509 and 368 ions with various *m/z* values were extracted from the BPCs in positive and negative modes, respectively. The peak areas of each ion in the extracted ion chromatograms were used to represent the quantities of the corresponding constituents while the comparisons were made between samples. In score plots of PCA, using all ions detected in both positive and negative modes, the general profiles of *D. nobile* grown for 1, 2, and 3 years were observed, except a couple of outliers (Figure 1E). The locations of the three clusters representing *D. nobile* samples of different ages were distributed along with component 1, indicating that the constituents with larger loading values in component 1 were more affected by age. Thus, based on the chromatograms and the PCA results, the metabolome of *D. nobile* stems was altered in an age‐dependent manner.

### 3.2 Discovery of the constituents in *D. nobile* stems of various ages

**FIGURE 2:**
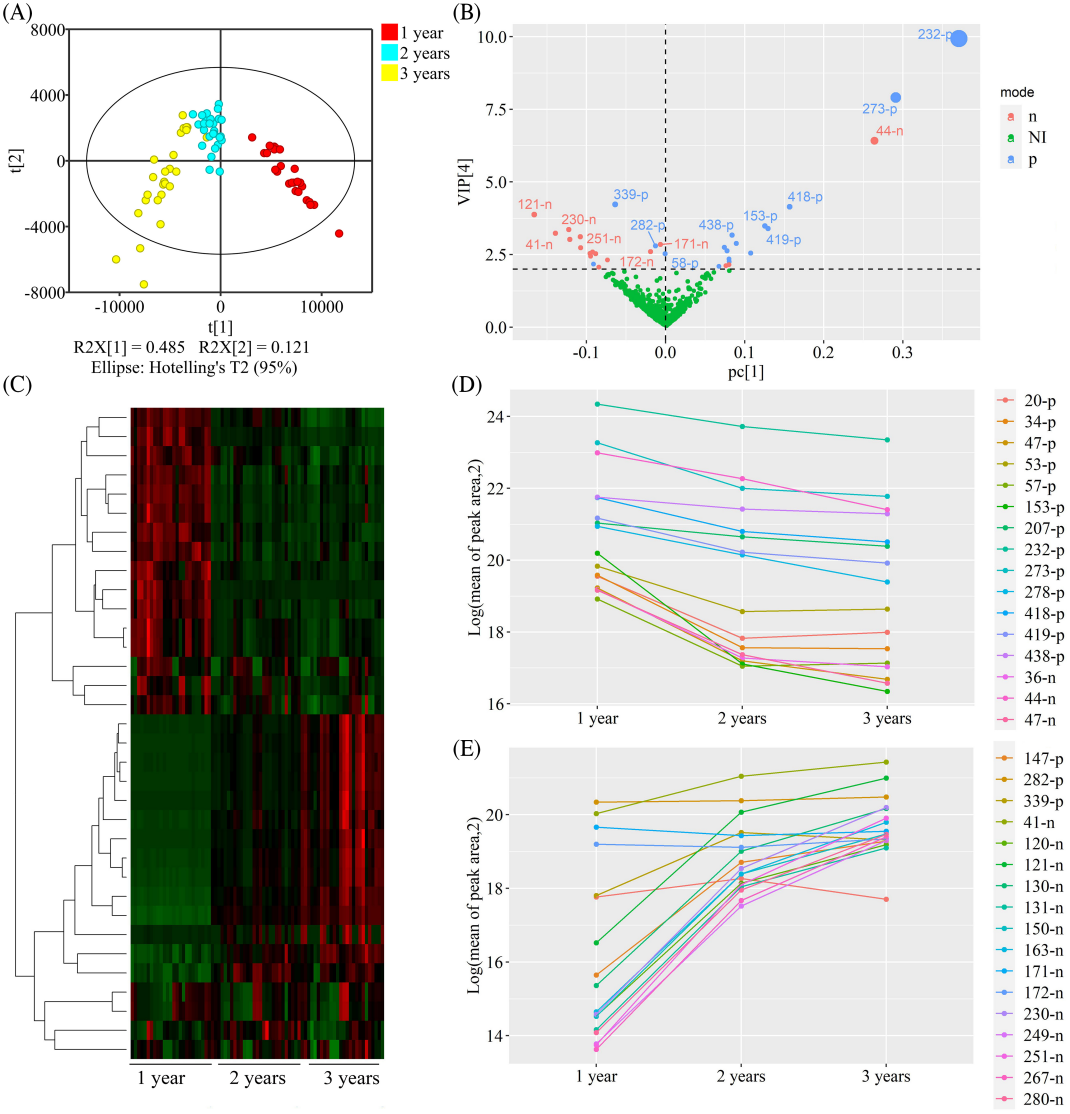
The constituents in *D. nobile* stems with an age‐dependent pattern. (A) Score plot of PLS‐DA based on the UPLC‐Q‐TOF/MS data of *D. nobile* from 1 to 3 years (R2X (cum) = 0.709, Q2 (cum) = 0.789). (B) VIP score plot of all metabolites in *D. nobile* in different growth years. Each point represents a metabolite. The dotted black line represents the VIP score value of 2. (C) One‐way hierarchical clustering analysis heatmap of 34 potential makers in all samples cultivated for 1, 2, and 3 years. (D) Line chart of 16 potential markers for which the peak area decreased with age. (E) Line chart of 18 potential markers for which the peak area increased with age. P, positive ion mode; N, negative ion mode

To screen the constituents that can be used to distinguish between samples of various ages, the outliers observed in the score plots of PCA were removed from the data, and a PLS‐DA was performed and passed the permutation tests. In the score plot of PLS‐DA (Figure 2A), points representing samples from different planting years were located away from each other, while points representing samples with the same ages were clustered together. In addition, the PLS‐DA model also had a good predictive ability and credibility: R2Y = 0.709; Q2 (cum) = 0.789. To find out which contents were significantly different among *D. nobile* stems aged 1, 2, and 3 years, a variable importance in projection (VIP) plot was generated (Figure 2B). In the VIP plot, in total 34 constituents with VIP scores of more than 2 were selected (Table 2). Most constituents detected in positive mode were distributed on the right side of the plots, which meant that their levels may be high in 1 ‐year‐old *D. nobile* stems. In contrast, most points located on the left side of the VIP plots, representing highly expressed constituents in 3‐year‐old *D. nobile* stems, were detected by MS in negative mode. To confirm this finding, a one‐way hierarchical clustering analysis heatmap was generated by using data of 34 constituents with PLS‐DA VIP scores of more than 2. Based on the expression pattern along the planting years, the constituents were generally clustered into two groups (Figure 2C). The contents of 16 out of 34 constituents (cluster 1) decreased over time (Figure 2D), while the contents of 18 out of 34 constituents (cluster 2) increased over time (Figure 2E). Most constituents in cluster 1 were observed by MS in positive mode, whereas most constituents in cluster 2 were detected by MS in negative mode, confirming our PCA results.

**TABLE 2:** The information of 34 markers from *D. nobile* samples

ID	VIP	Rt (min)	Add ion	*m*/*z* actual	*m*/*z* theoretical	Molecular formula	Identification	MS/MS
232‐p	9.93	3.09	[M+H]^+^	264.1969	264.1958	C_16_H_25_NO_2_	Dendrobine	218.1910; 176.1441; 105.0699; 70.0655; 145.1014;
273‐p	7.90	3.32	[M+H]^+^	280.1917	280.1907	C_16_H_25_NO_3_	2‐Hydroxydendrobine or 6‐hydroxydendrobine	263.1884; 220.1326; 136.1118;
44‐n	6.41	0.60	[M−H]^−^	191.0567	191.0561	C_7_H_12_O_6_	Quinic acid	127.0399; 93.0343; 85.0295;
339‐p	4.22	3.69	[M+H]^+^	292.1902	292.1907	C_17_H_25_NO_3_	Dendroxine	248.2001; 205.1445; 178.1210; 93.069; 56.0490;
418‐p	4.15	2.29	[M+H]^+^	264.1954	264.1958	C_16_H_25_NO_2_	Isomer of dendrobine	176.1441; 122.0970;
121‐n	3.88	2.75	[M−H]^−^	445.2073	445.2079	C_21_H_34_O_10_	Dendromoniliside D or isomer	321.1341; 265.1450; 161.0461; 101.0239; 71.0134;
153‐p	3.48	2.82	[M+H]^+^	262.1799	262.1802	C_16_H_23_NO_2_	Unknown	95.0854;
419‐p	3.40	4.22	M^+^	332.2597	332.2584	C_21_H_34_NO_2_^+^	*N*‐isopentenyl‐dendrobinium	264.1973; 176.1435; 69.0704;
230‐n	3.36	3.51	[M−H]^−^	559.2750	559.2760	C_27_H_44_O_12_	Dendronobiloside C/D or isomer	517.0897; 298.0759; 179.0552; 119.0338; 89.0239; 71.0142;
41‐n	3.23	0.62	[M−H]^−^	341.1085	341.1089	C_12_H_22_O_11_	Sucrose	89.0240; 71.0135; 59.0135
438‐p	3.17	4.53	M^+^	292.1919	292.1907	C_17_H_26_NO_3_^+^	Fragment of *N*‐isopentenyl‐dendroxinium	NA
251‐n	3.11	3.51	[M−H]^−^	559.2750	559.2760	C_27_H_44_O_12_	Dendronobiloside C/D or isomer	517.0897; 298.0759; 179.0552; 119.0338; 89.0239; 71.0142;
130‐n	3.02	2.83	[M−H]^−^	445.2070	445.2079	C_21_H_34_O_10_	Dendromoniliside D or isomer	413.2164; 360.1420; 283.1530; 221.1526; 161.0424; 101.0245;
34‐p	2.88	0.68	[M+H]^+^	262.1286	262.1285	C_11_H_19_NO_6_	Isomer of succinylcarnitine	244.1183; 216.1239; 198.1128; 72.0807;
171‐n	2.85	3.09	[M−H]^−^	577.1565	577.1563	C_27_H_30_O_14_	Violanthin	533.1282; 503.1175; 457.1128; 383.0769; 353.0652; 191.0315;
282‐p	2.80	3.42	[M+H]+	306.2073	306.2064	C_18_H_27_NO_3_	1H‐Cyclopent[cd]indole‐5‐carboxylic acid, decahydro‐1,7b‐dimethyl‐7‐methylene‐6‐(1‐methylethyl)‐2‐oxo‐, methyl ester, (2aa,4aa,5b,6a,7aa,7ba) ‐ (9CI), malonyl‐L‐carnitine or isomer	292.1860; 263.1867; 233.0591; 175.1476; 133.1012; 107.0852;
20‐p	2.75	0.60	[M+H]^+^	248.1132	248.1129	C_10_H_17_NO_6_	Isomer of malonyl‐l‐carnitine	230.1028; 182.0819; 116.0710; 98.0597; 87.0439;
163‐n	2.73	3.02	[M−H]^−^	445.2073	445.2079	C_21_H_34_O_10_	Dendromoniliside D or isomer	413.2148; 281.1389; 221.1548; 162.0436; 89.0240; 59.0133;
53‐p	2.62	0.93	[M+H]^+^	276.1447	276.1442	C_12_H_21_NO_6_	Unknown	258.1335; 230.1391; 86.0962;
172‐n	2.60	3.14	[M−H]^−^	577.2012	577.2079	C_32_H_34_O_10_	Unknown	NA
267‐n	2.58	3.77	[M−H]^−^	559.2750	559.2760	C_27_H_44_O_12_	Dendronobiloside C/D or isomer	536.0630; 457.1714; 179.0559; 119.0347; 89.0244; 59.0135;
278‐p	2.55	3.43	[M+H]^+^	294.2068	294.2064	C_17_H_27_NO_3_	Nobilonine	249.1491; 203.1425; 175.1484; 95.085;
280‐n	2.54	3.91	[M−H]^−^	563.3060	563.3073	C_27_H_48_O_12_	Dendronobiloside A	401.2472; 113.0229; 101.0231; 71.0131; 59.0131;
58‐p	2.53	1.33	M^+^	103.0540	103.0542	C_8_H_7_^+^	Unknown	91.0540; 77.0386; 51.0228;
249‐n	2.52	3.71	[M−H]^−^	561.2871	561.2917	C_27_H_46_O_12_	Unknown	399.2333; 161.0429; 101.0232; 59.0129;
150‐n	2.44	2.98	[M−H]^−^	413.2173	413.2181	C_21_H_34_O_8_	Dendronobiloside E/Dendromoniliside A	350.1051; 293.1501; 265.1447; 216.1370; 101.0241; 59.0132;
207‐p	2.35	3.00	M^+^	278.2118	278.2115	C_17_H_28_NO_2_^+^	*N*‐Methyl‐dendrobinium	250.2194; 220.1355; 187.0016; 133.1005; 105.0689; 84.0807;
120‐n	2.31	2.81	[M−H]^−^	445.1319	445.1351	C_19_H_26_O_12_	Unknown	NA
47‐p	2.26	0.88	[M+H]^+^	300.1441	300.1442	C_14_H_21_NO_6_	Unknown	NA
147‐p	2.18	2.82	[M+H]^+^	469.1355	469.1341	C_21_H_24_O_12_	Unknown	NA
47‐n	2.15	0.64	[M−H]^−^	137.0238	137.0244	C_7_H_6_O_3_	Protocatechualdehyde	NA
36‐n	2.12	0.61	[M−H]^−^	357.1026	357.1038	C_12_H_22_O_12_	Lactobionic acid or isomer	173,0448; 89.0242;
57‐p	2.10	1.30	[M+H]^+^	310.1283	310.1285	C_15_H_19_NO_6_	2‐Methyl‐1H‐indol‐7‐yl‐β‐d‐mannopyranoside or isomer	292.1179; 264.1227; 120.0809;
131‐n	2.07	2.88	[M−H]^−^	479.1686	479.1685	C_23_H_24_N_6_O_6_	Unknown	NA

Unknown means the compound is not found in *Dendrobium*.

### 3.3 Assignment of the general structures of constituents showing age‐dependent expression in *D. nobile* stems

**FIGURE 3:**
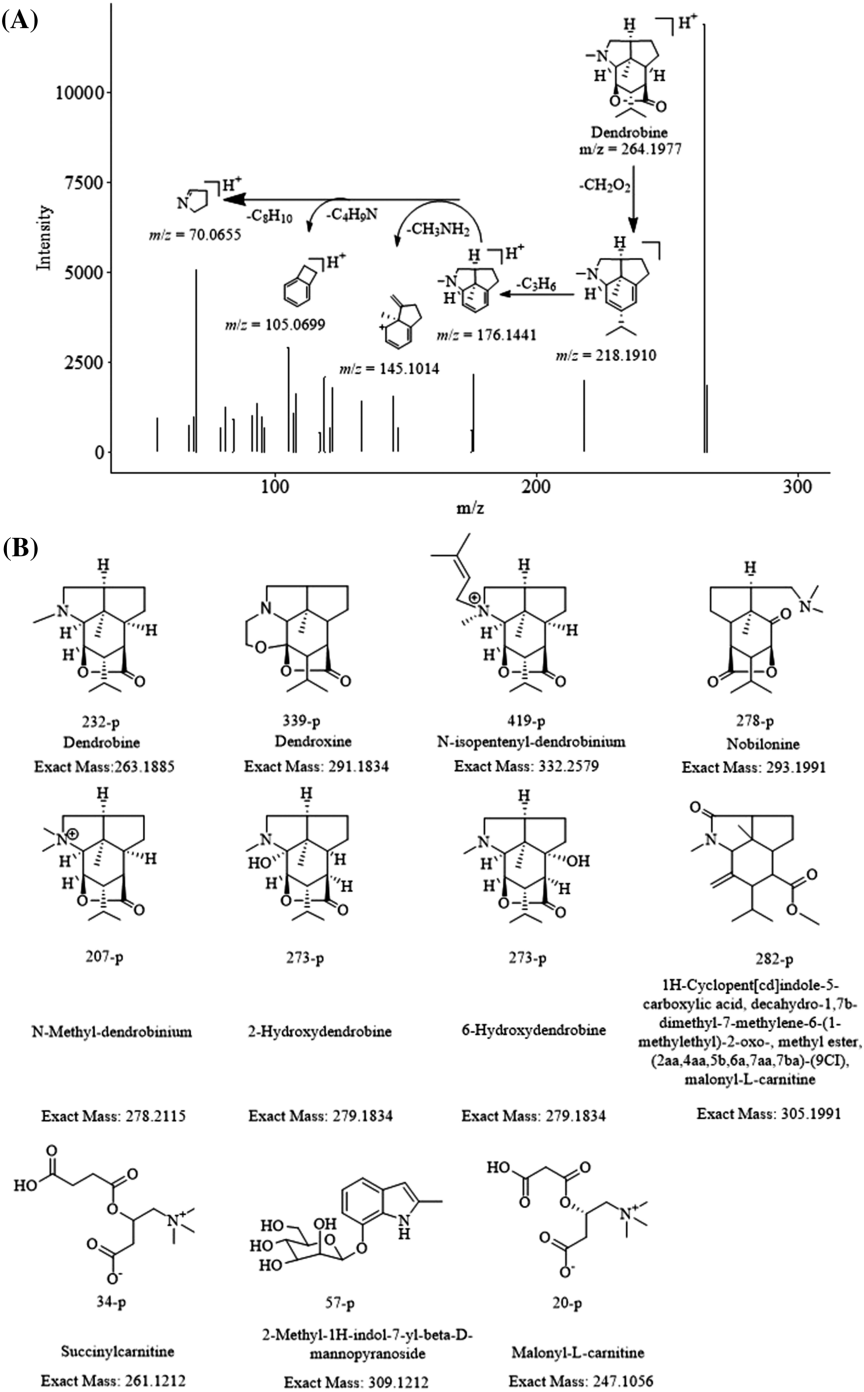
The chemical structure of compounds contributing to the discrimination of *D. nobile* in different growth years. (A) Targeted MS/MS and fragmentation pattern of dendrobine. (B) The chemical structure of the typical compounds identified in positive ion mode [Correction added on 20 April 2022, after first online publication: Figure 3 has been corrected.]

To identify the structures or structural features of the 34 constituents with age‐dependent expression in *D. nobile* stems, information including the exact molecular weight, fragment ions in MS, chromatographic retention time, reference literature, and reference compounds was integrated. Before the structure identification, as much information of compounds reported in *D. nobile* was collected as possible. In the positive mode of MS, by matching the exact *m/z* values of quasi‐molecular ions with the exact molecular weights of the reported compounds in *D. nobile*, in total 17 structures were screened out. Most of those structures were potentially dendrobine‐type alkaloids. Furthermore, the chromatographic retention time of constituent #232‐p was identical to that of the reference compound dendrobine (Figure 3A). Constituents #339‐p, #419‐p, #278‐p, and #207‐p were identified as dendroxine, *N*‐isopentenyl‐dendrobinium, nobilonine, and *N*‐methyl‐dendrobinium by searching an in‐house library and comparing their formulae and fragmentation information with the literature.^11^ Constituents #273‐p and #418‐p were identified as the isomers 2‐hydroxydendrobine or 6‐hydroxydendrobine, isomer of dendrobine, by searching our in‐house library. However, the exact structure could not be confirmed due to a lack of reference standard or literature. Constituents #438‐p was deduced as in‐source fragments of *N*‐isopentenyl‐dendroxinium by comparison of the retention time and fragmentation information with their corresponding precursor ions. Constituents (1) #34‐p, (2) #282‐p, (3) #20‐p, and (4) #57‐p showed not only extremely similar exact molecular weights, but also similar fragmentation mechanisms as (1) succinylcarnitine, (2) 1H‐cyclopent[cd]indole‐5‐carboxylicacid, decahydro‐1,7b‐dimethyl‐7‐methylene‐6‐(1‐methylethyl)‐2‐oxo‐, methyl ester, (2aa,4aa,5b,6a,7aa,7ba) ‐ (9CI), malonyl‐l‐carnitine, (3) malonyl‐L‐carnitine, and (4) 2‐methyl‐1H‐indol‐7‐yl‐β‐d‐mannopyranoside, respectively. However, although their general structural features were elucidated, these constituents could not be uniquely identified due to a lack of reference compounds (Figure 3B). As 2‐ and 6‐hydroxydendrobine have been reported previously in *D. nobile* stems, we tentatively identify constituent #273‐p as 2‐ or 6‐hydroxydendrobine. As the structure of dendrobine had already been uniquely assigned to constituent #232‐p, the link between constituent #418‐p and dendrobine was broken. However, we still assume it is a dendrobine isomer based on its general structural features and our in‐house library. In the same way, constituents (1) #34‐p, (2) #282‐p, (3) #20‐p, and (4) #57‐p were accordingly identified as (isomers of) (1) succinylcarnitine, (2) 1H‐cyclopent[cd]indole‐5‐carboxylic acid, decahydro‐1,7b‐dimethyl‐7‐methylene‐6‐(1‐methylethyl)‐2‐oxo‐, methyl ester, (2aa,4aa,5b,6a,7aa,7ba) ‐ (9CI), malonyl‐l‐carnitine, (3) malonyl‐L‐carnitine, and (4) 2‐methyl‐1H‐indol‐7‐yl‐β‐d‐mannopyranoside, respectively. The exact molecular weights of constituents #153‐p, #53‐p, #58‐p, #47‐p, and #147‐p matched nothing in the in‐house library; however, based on the predicted molecular formulae, they could be identified as alkaloids although their exact structures remain unknown. As their expression followed the positive age‐dependent patterns as observed for dendrobine, dendroxine, *N*‐isopentenyl‐dendrobinium, nobiline, and *N*‐methyl‐dendrobinium, we also assumed they are dendrobine‐type alkaloids.

**FIGURE 4:**
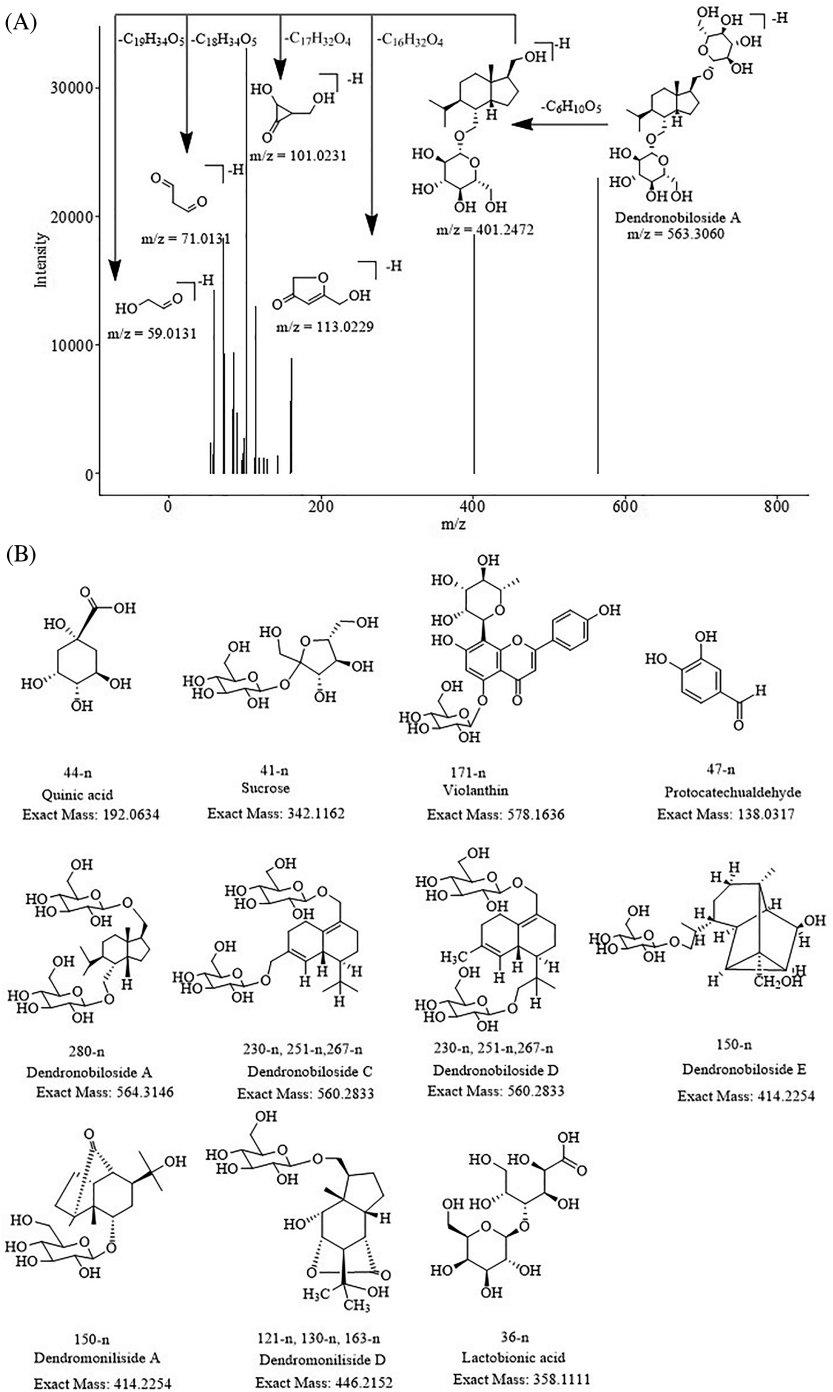
The targeted MS/MS and chemical structure of compounds contributing to the discrimination of *D. nobile* in different growth years. (A) Targeted MS/MS and fragmentation pattern of dendronobiloside A. (B) The chemical structure of the typical compounds identified in negative ion mode

In the negative mode of MS, 17 constituents with PLS‐DA VIP scores of more than 2 were screened out. In the same way as above, using the exact molecular weight values, ion fragmentation mechanisms, and the information of reference compounds, constituents #44‐n, #41‐n, #171‐n, and #47‐n were uniquely identified as quinic acid, sucrose, violanthin, and protocatechualdehyde, respectively. By matching the exact molecular weights with members of our in‐house library, constituents #121‐n, #230‐n, #251‐n, #130‐n, #163‐n, #267‐n, #280‐n, and #150‐n were identified as dendronobilosides or dendromonilisides (Figure 4B). Their MS/MS fragment ions were also linked to parts of the predicted structures. Among those constituents, #280‐n was uniquely identified as dendronobiloside A based on their formulae and fragmentation information obtained from the literature (Figure 4A).^9^ Others were only identified as isomers of their corresponding predicted structures because of the lack of corresponding reference compounds. Constituent #36‐n was tentatively identified as one of the isomers of lactobionic acid. Regarding constituents #172‐n, #249‐n, #120‐n, and #131‐n, the exact molecular weight values matched nothing in our in‐house library or any reference standards. Therefore, their structures remain unknown.

### 3.4 Statistics of the structure‐confirmed constituents with age‐dependent expression and validation with dendrobine quantification

**FIGURE 5:**
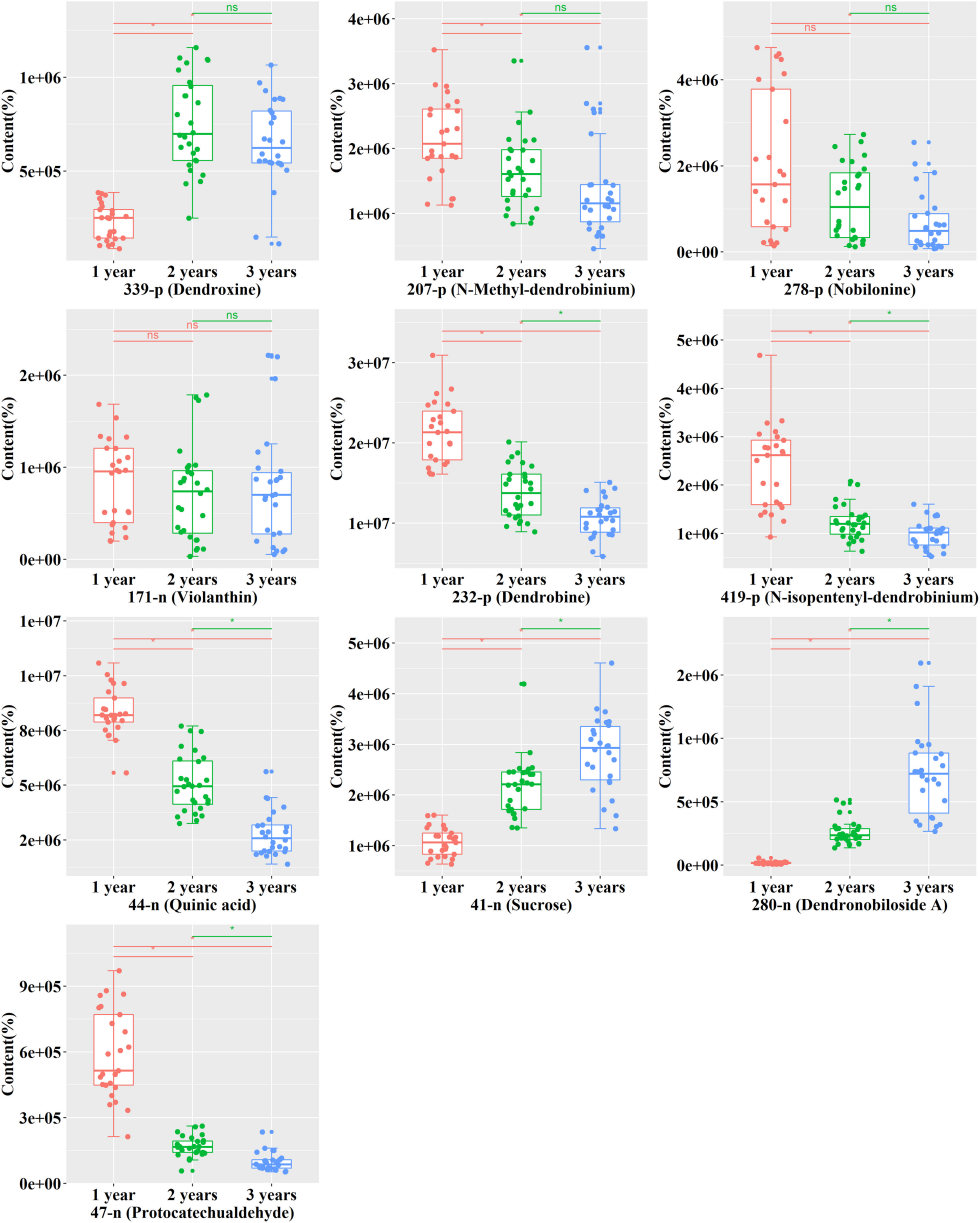
The relative content of identified compounds in the stems of *D. nobile* in different growth years. 339‐p: dendroxine; 207‐p: *N*‐methyl‐dendrobinium; 278‐p: nobilonine; 171‐n: violanthin; 232‐p: dendrobine; 419‐p: *N*‐isopentenyl‐dendrobinium; 44‐n: quinic acid; 41‐n: sucrose; 280‐n: dendronobiloside A; 47‐n: protocatechualdehyde. *P < 0.05; ns, no significant difference

To find a marker compound to determine the age of *D. nobile*, the identification of one or multiple structure‐confirmed constituents with an age‐dependent expression pattern is important. Among all constituents with age‐dependent expression patterns, #232‐p, #339‐p, #419‐p, #278‐p, and #207‐p, which were detected in positive mode, were uniquely identified as dendrobine, dendroxine, *N*‐isopentenyl‐dendrobinium, nobiline, and *N*‐methyl‐dendrobinium, respectively; #44‐n, #41‐n, #171‐n, #280‐n, and #47‐n, which were detected in negative mode, were uniquely identified as quinic acid, sucrose, violanthin, dendronobiloside A, and protocatechualdehyde, respectively. The statistical significance of the age‐dependent expression of these structure‐confirmed constituents was analyzed by one‐way ANOVA followed by Tukey's test (Figure 5). Constituents #339‐p and #207‐p passed the test between year 1 and year 2 and between year 1 and year 3; however, they failed to pass the test between year 2 and year 3. Constituent #278‐p merely passed the test between year 1 and year 3. Moreover, constituent #171‐n failed to pass the test between any two years. Constituents #232‐p, #419‐p, #44‐n, #41‐n, #280‐n, and #47‐n passed the test between any two years, thus showing an age‐dependent expression pattern not only based on the visualized data but also at a statistically significant level. Thus, the above constituents can be used as markers to distinguish between *D. nobile* of different age.

**FIGURE 6:**
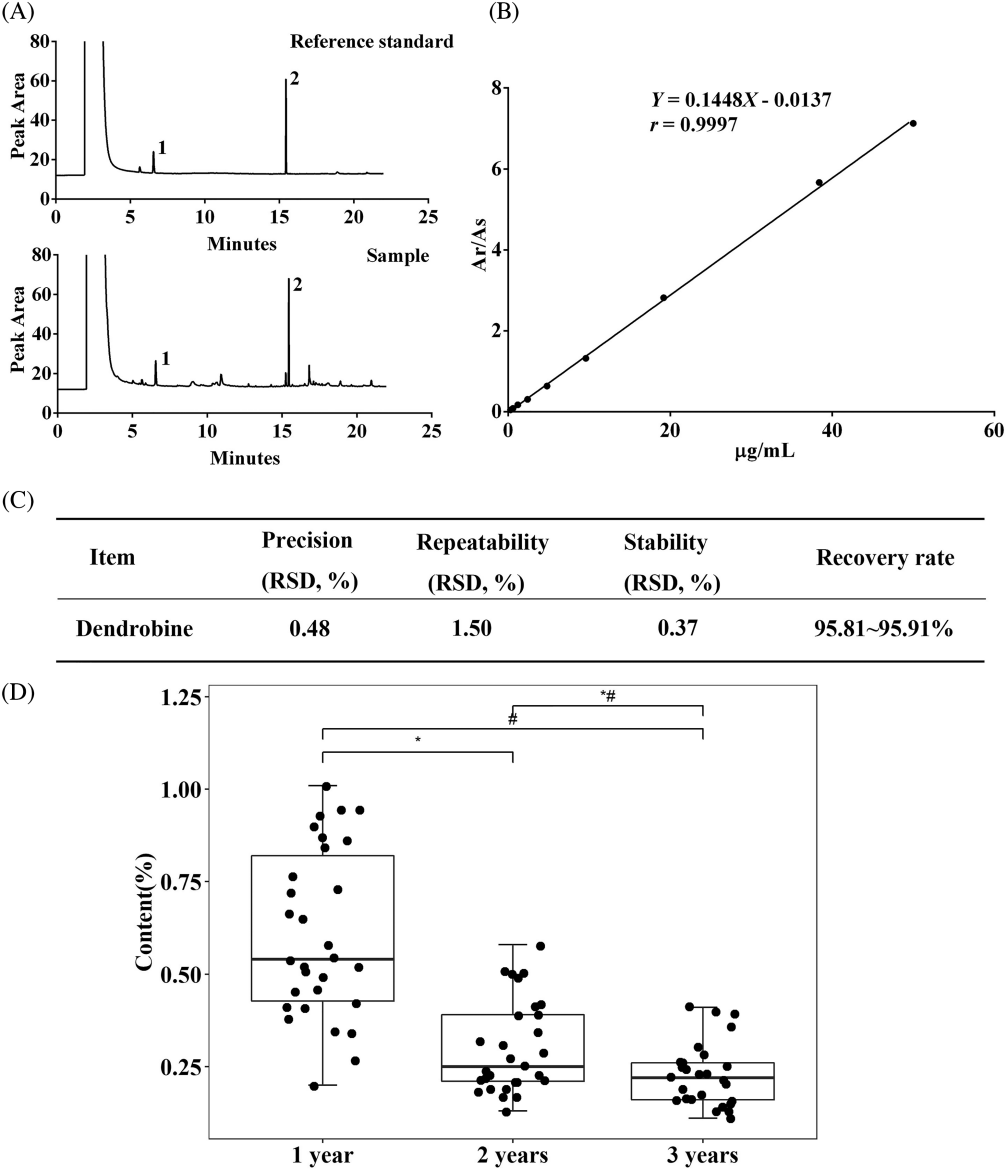
Validation of the quantification method and the effect of age on dendrobine content. (A) Gas chromatograms of a reference standard and a typical sample. (B) Calibration curve of 0.30–50.00 μg/mL dendrobine. (C) The precision, repeatability, stability, and recovery rate of the method. (D) The dendrobine content in the stems of *D. nobile* of different age. *P < 0.05 comparing year 2 to year 1; ^#^ *P* < 0.05 comparing year 3 to year 1; ^#^*P < 0.05 comparing year 3 to year 2

The absolute quantification of dendrobine was used to validate the statistical analysis results using peak areas. This quantification method was established by other institutions and was validated in our group. As shown in Figure 6A, dendrobine and naphthalene did not interfere with each other under this detection condition. The retention time of dendrobine in the sample solution is the same as that of the reference standard solution, and there is no background interference. The separation coefficient of dendrobine from adjacent peaks was greater than 1.5, indicating that the method was specific. The correlation coefficient of the standard curve was 0.9997, indicating that dendrobine has a good linear relationship in the linear range of 0.30–50.00 μg/mL (Figure 6B). The recovery rate (95.81–95.91%), relative standard deviation (RSD) between injections (0.48%), RSD between samples (1.50%), and RSD between samples stored for various durations (0.37%) showed that the method was accurate, precise, stable, and reliable (Figure 6C). In agreement with the MS data, the GC data confirmed that dendrobine content in *D. nobile* decreased with age. The difference of dendrobine content between two groups was statistically significant (*P* < 0.05, Figure 6D), confirming the MS results.

### 3.5 The reason behind the trends of glycosides and alkaloids in *D. nobile* of different age

In most plants, the levels of glycosides of naturally existing molecules increase as plants get older. The most well‐known example is the saponin family. Saponins are glycosides of triterpenes with four rings.^14^ This family is biosynthesized in various well‐known medicinal plants,^15^, ^16^, ^17^ and saponins are expressed in an age‐dependent manner in *Panax ginseng*, *Panax notoginseng*, and *Polygala tenuifolia*.^18^, ^19^, ^20^ Another famous glycoside family is formed by the flavonoid glycosides, which also exhibit a positively age‐dependent expression pattern.^21^ Even in some regular food sources, small molecules with very simple structures, like isothiocyanates in curly kale, follow the same pattern.^22^ Thus, it is not surprising that positively age‐dependent expression patterns of glycosides were observed in the present study.

However, for alkaloids, completely opposite observations were made in *D. nobile*. Alkaloids are also expressed in a broad range of plants.^23^ Generally, alkaloid contents increase while plants grow. Examples include cinchonine, cinchonidine, quinidine, and quinine in *Cinchona ledgeriana* Moens.,^24^ as well as galanthamine, lycorine, and lycoramine in *Lycoris chinensis*.^25^ However, in the case of dendrobine‐type alkaloids in *D. nobile*, with increasing age, the contents decreased. This may be linked to the plant defense system. *Dendrobium* spp. possess aerial roots and require microorganisms to support nutrition.^26^ Thus, while utilizing microorganisms, there is a need to prevent attacks by phytopathogenic microbes during plant development. This hypothesis is partially supported by another study regarding dendrobine in a microbe named *Trichoderma longibrachiatum*. In addition to *D. nobile*, dendrobine was also detected in this microorganism. Dendrobine‐containing *T. longibrachiatum* showed much stronger anti‐bacterial activity than other similar microbes without dendrobine.^27^ Possibly, dendrobine protects young *D. nobile* against phytopathogenic microbes, while this might not be necessary in older *D. nobile*, which have a mature immune system. This speculation remains to be confirmed by future experiments.

With increasing age, the increase of *Dendrobium*‐specific glycosides and the decrease of dendrobine‐type alkaloids in *D. nobile* may be related. Dendrobine is one of the typical structures of sesquiterpene alkaloids.^28^ Dendrobine has only been isolated from *D. nobile*, while dendrobine‐type alkaloids have been detected in various *Dendrobium* species. The structures of dendrobine‐type alkaloids are complex; thus, multiple biosynthesis pathways may exist. A study published in 2017 proposed a biosynthesis scheme of dendrobine.^29^ In this scheme, an oxide metabolite of copacamphane was considered as one of the mandatory intermediate compounds for dendrobine biosynthesis in *D. nobile*. The glycone of constituent #150‐n (dendromoniliside A) in the present study is just the hydroxylated product of this intermediate. Similarly, in the aforementioned scheme, one of the intermediates, the mono‐oxidative metabolite of the dehydrated picrotoxane, is just the dehydrogenated product of the glycone of dendronobiloside A that was assigned to constituent #280‐n in the present study. Other than constituents #150‐n, #280‐n, constituents #121‐n, #230‐n, #251‐n, #130‐n, #163‐n, and 267‐n were also identified as dendronobilosides or dendromonilisides. All these aforementioned constituents share similar glycone structures that are also similar to intermediates in the biosynthesis pathway of dendrobine, while also being negatively correlated with dendrobine‐type alkaloid accumulation in *D. nobile*. Thus, we hypothesize that glycosylation of intermediates in dendrobine‐type alkaloid biosynthesis pathways may play a competitive role in the generation of dendrobine‐type alkaloids in *D. nobile*.

The opposite accumulation trends seem to be observed just between dendrobine‐type alkaloids and *Dendrobium*‐specific glycosides, including dendronobilosides and dendromonilisides. A flavonoid glycoside named violanthin, assigned to constituent #171‐n, was not influenced by the age of *D. nobile*, maybe due to the low degree of crosstalk between biosynthesis pathways of flavonoids and dendrobine‐type alkaloids.^30^ Constituent #57‐p is a special glycoside which was detected by MS in positive mode. This constituent was tentatively identified as the glycoside of a derivative of indole alkaloids and showed even a reversed expression pattern in contrast to dendronobilosides and dendromonilisides. A study published in 2020 proposed an alternative biosynthesis pathway of dendrobine, in which indole alkaloid derivatives play an important role.^31^ The shikimate pathway is upstream of the indole alkaloid pathway.^32^ Meanwhile, shikimate is biosynthesized from 3‐dehydroquinic acid, which is a major metabolite of quinic acid.^33^ In the present study, we observed a decreasing trend of quinic acid during *D. nobile* growth. This age‐dependent decreasing expression pattern of quinic acid was not only observed in the present study but also in several other plant species.^34^ Thus, taken together, the substrate‐competitive effect from the generation of dendronobilosides and dendromonilisides and the substrate‐reductive effect from the decreased synthesis of quinic acid and indole alkaloids may work together to contribute to the decreasing trend of dendrobine during *D. nobile* growth.

Dendrobine‐type alkaloids share a sesquiterpene backbone that was verified to be synthesized mainly via the mevalonate pathway and the methyl‐d‐erythritol 4‐phosphate pathway.^29^ In the two pathways, 16 key enzymes are involved. Among 16 key enzymes involved in terpenoid backbone biosynthesis, the expression levels of the genes encoding acetyl‐CoA C‐acetyltransferase (AACT), 1‐deoxy‐d‐xylulose‐5‐phosphate synthase (DXS), 4‐hydroxy‐3‐methylbut‐2‐en‐1‐yl diphosphate synthase (HDS), geranyl diphosphate synthase (GPPS), 1‐deoxy‐d‐xylulose‐5‐phosphate reductoisomerase (DXR), and 3‐hydroxy‐3‐methylglutaryl coenzyme A reductase (HMGR) were different between plants in different stages, including ginger rhizome, *Achillea millefolium*, and *Solanum tuberosum* L. The expression levels of AACT, DXS, and HDS in young ginger rhizome were higher than in mature ones.^35^ Moreover, the RT‐PCR results show that the expression levels of genes encoding DXR and GPPS in leaves of *A. millefolium* at different developmental stages were also different; young leaves exhibit higher expression levels of the above genes than fully expanded leaves.^36^ HMGR enzyme activity showed the same trend.^37^ The above results are in agreement with the observation that the content of alkaloids in 1‐year‐old *D. nobile* stems is higher than that in 2‐ and 3‐year‐old stems. However, over time, sesquiterpenes are also glycosylated. Glycosylation of plant secondary metabolites, including terpenoids, flavonoids, and other small molecules, is mainly catalyzed by UDP‐glucuronosyltransferases (UGTs).^38^ Previous studies have shown that the relative mRNA expression levels of UGTs are also different between different growth stages of plants. Additionally, the relative expression levels of UGTs, including UGTSr, UGFT, UGTs (UGT71A33, UGT71A35, UGT71A34, UGT71W2, and UGT75T1), were confirmed to be higher in the mature stage than in the young stage in *Stevia rebaudiana*, litchi, and strawberry.^39^, ^40^, ^41^ Therefore, we deduced that the above enzymes, which are involved in the synthesis of alkaloids and glycosides, are among the major causes of the opposite trend observed for the two main compounds in *D. nobile*.

## 4 CONCLUSION

In the present work, the metabolites in *D. nobile* stems were analyzed using UPLC‐ Q/TOF‐MS with multivariate statistical analysis. The results reveal that trends of alkaloids and sesquiterpene glycosides in the stems of *D. nobile* in different growth years were the opposite. The contents of alkaloids in stems were found to decrease with age, whereas the content of sesquiterpene glycosides follows an opposite trend. Furthermore, the absolute quantification of dendrobine confirmed the increasing trend of alkaloids. Combining our results with the pharmacological effects of the above chemical components, we recommend to use 1‐year‐old *D. nobile* for clinical treatment of neurological diseases, while 3‐year‐old *D. nobile* could be used to improve immunity. Finally, sesquiterpene glycosides might be quality‐related constituents that could be used as quality markers in *D. nobile*.

## Data Availability

The datasets used and/or analysed during the current study are available from the corresponding author on reasonable request.
